# Induction Motor Fault Diagnosis Using Support Vector Machine, Neural Networks, and Boosting Methods

**DOI:** 10.3390/s23052585

**Published:** 2023-02-26

**Authors:** Min-Chan Kim, Jong-Hyun Lee, Dong-Hun Wang, In-Soo Lee

**Affiliations:** School of Electronics Engineering, Kyungpook National University, Daegu 41566, Republic of Korea

**Keywords:** induction motor, fault diagnosis, support vector machine, multilayer neural network

## Abstract

Induction motors are robust and cost effective; thus, they are commonly used as power sources in various industrial applications. However, due to the characteristics of induction motors, industrial processes can stop when motor failures occur. Thus, research is required to realize the quick and accurate diagnosis of faults in induction motors. In this study, we constructed an induction motor simulator with normal, rotor failure, and bearing failure states. Using this simulator, 1240 vibration datasets comprising 1024 data samples were obtained for each state. Then, failure diagnosis was performed on the acquired data using support vector machine, multilayer neural network, convolutional neural network, gradient boosting machine, and XGBoost machine learning models. The diagnostic accuracies and calculation speeds of these models were verified via stratified K-fold cross validation. In addition, a graphical user interface was designed and implemented for the proposed fault diagnosis technique. The experimental results demonstrate that the proposed fault diagnosis technique is suitable for diagnosing faults in induction motors.

## 1. Introduction

Induction motors are an important component in the industries. These motors are mechanically robust; therefore, the surrounding environment does not affect their operation [1]. However, their long-term use can lead to the deformation and wear of parts, thereby reducing the life and affecting the performance of induction motors. A fault occurring in an induction motor can lead to material damage, time wastage, and workplace accidents, reducing overall system reliability [2,3]. Thus, a fault diagnosis technology that can detect faults early, take appropriate action, and ensure equipment safety and reliability is required.

Induction motor faults are classified into electrical and mechanical [4]. Electrical faults include overload, open-phase, and short-circuit faults, while mechanical faults include bearing, rotor, and stator faults [5]. Notably, bearing faults are frequent and account for approximately 40% of all faults [6].

Previous fault diagnosis techniques are based on mathematical models of the system and user reviews [7,8]. However, obtaining an accurate mathematical model of a system is difficult because of its nonlinearities. Methods based on user reviews utilize visual and auditory information obtained by observing the equipment, resulting in unnecessary costs due to over-maintenance and sudden faults [9].

Thus, machine-learning-based fault diagnosis techniques have been recently proposed. As machine learning models can learn the data of nonlinear systems, fault diagnosis techniques based on machine learning can learn the data without expert-level knowledge of the equipment.

Fault diagnosis techniques based on machine learning models often use specific signals such as vibration, sound, current, and thermal images. Alternatively, data can be converted to another format and then feature extracted, as in [10], or neural networks can be used to perform fault diagnosis using temporal and spatial features of the data, as in [11]. Fault diagnosis techniques using electrical signals are often limited to electrical faults and are not suitable for mechanical engines. Techniques using thermal imaging can identify failure areas and are noninvasive, but they take time to measure temperature changes. Techniques using acoustic signals are cheaper than the other methods and perform well for certain mechanical faults and are noninvasive. However, acoustic signals are easily interfered with by ambient noise [12,13].

In this study, we obtained vibration data from an induction motor and performed fault diagnosis for bearing and rotor faults. Herein, a simulator was constructed using induction motors with normal, bearing fault, and rotor fault states to obtain vibration data. After obtaining the vibration data, stratified K-fold cross validation was conducted using support vector machine (SVM), multilayer neural network (MNN), convolutional neural network (CNN), gradient boosting machine (GBM), and XGBoost; the calculation speeds of these models were compared. Then, a graphical user interface (GUI) was implemented so that users can effectively diagnose the faults of induction motors.

## 2. Data Acquisition

**Figure 1:**
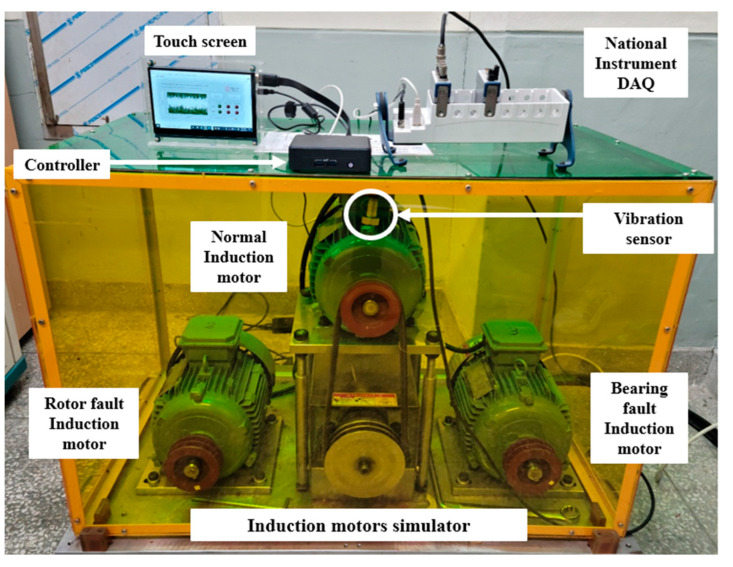
Experimental laboratory setup.

**Figure 2:**
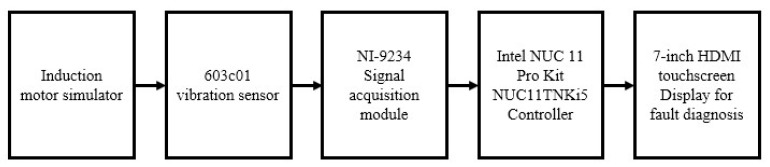
Block diagram of the experimental laboratory setup.

The experimental laboratory setup for data collection and induction motor fault diagnosis is shown in Figure 1, and Figure 2 shows the block diagram of Figure 1. The induction motor simulator comprised an induction motor in normal, rotor fault, and bearing fault states. Vibration data were collected using a 603C01 vibration sensor (IMI Sensors, NY, USA) and an NI-9234 signal acquisition module (National Instruments, TX, USA). The collected vibration data were used to train the SVM, MNN, CNN, GBM, and XGBoost models and diagnose faults. An Intel NUC 11 Pro Kit NUC11TNKi5 (Intel, CA, USA), a mini PC, was used to easily secure the workspace and diagnose faults at low power. In addition, a 7-inch HDMI Display-C, with a touchscreen display, was used to handle the fault diagnosis GUI without a mouse. The specifications of the vibration sensor used for data acquisition and fault diagnosis are listed in Table 1.

**Figure 3:**
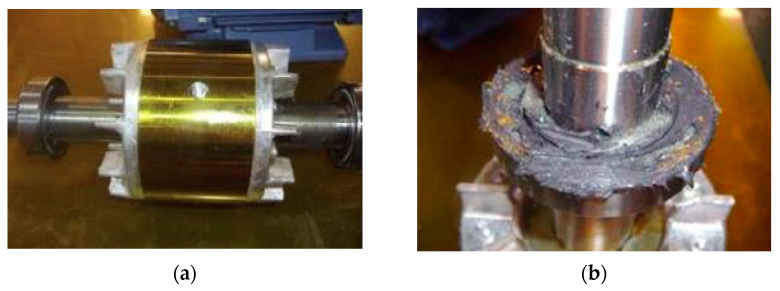
Induction motor fault type. (**a**) Rotor fault. (**b**) Bearing fault.

Figure 3 shows the faults in the induction motor. Figure 3a shows a rotor fault (i.e., a hole in the rotor bar), and Figure 3b shows a bearing fault (i.e., iron-powder presence in the bearing).

**Figure 4:**
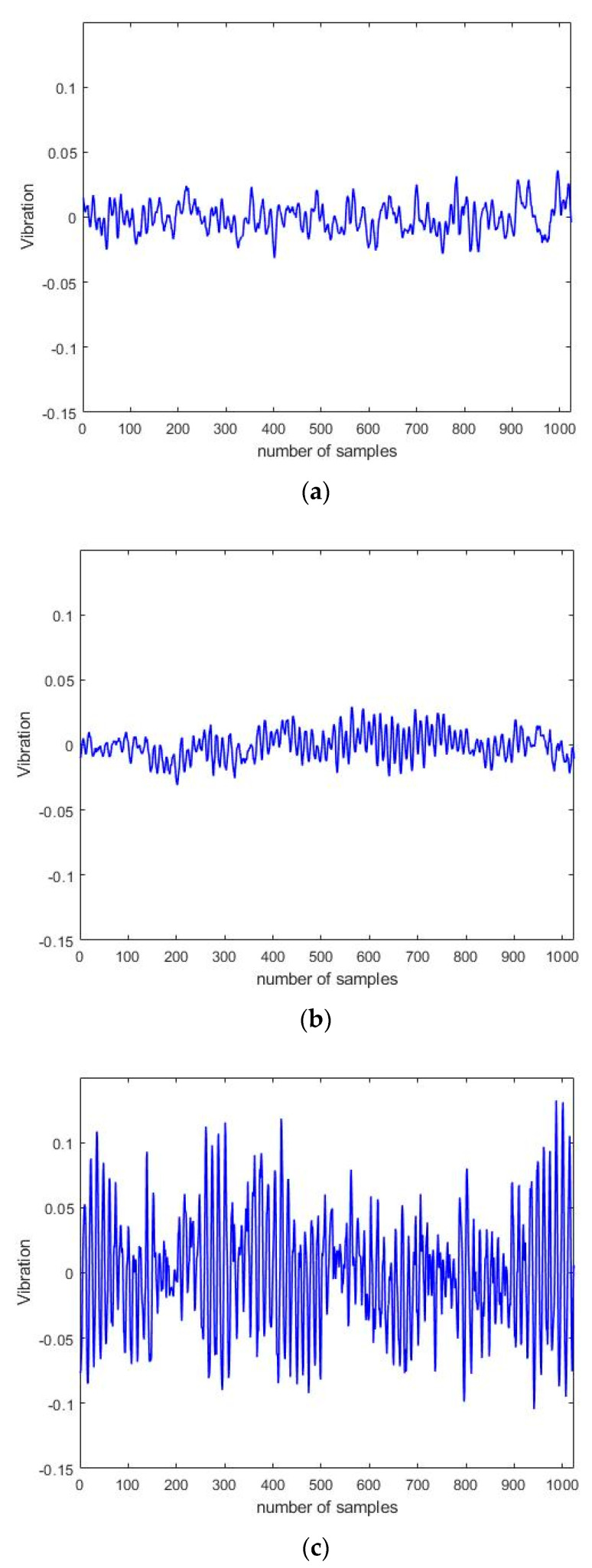
Data collected from the simulator. (**a**) Normal data. (**b**) Rotor fault data. (**c**) Bearing fault data.

Figure 4 presents a graph of a vibration dataset acquired using the induction motor simulator. There were 1240 datasets for each induction motor state; each dataset comprised 1024 data samples. The collected data were used to train and verify each of the compared models. Figure 4a–c show the normal, rotor fault, and bearing fault data, respectively.

## 3. Induction Motor Fault Diagnosis Algorithm

**Figure 5:**
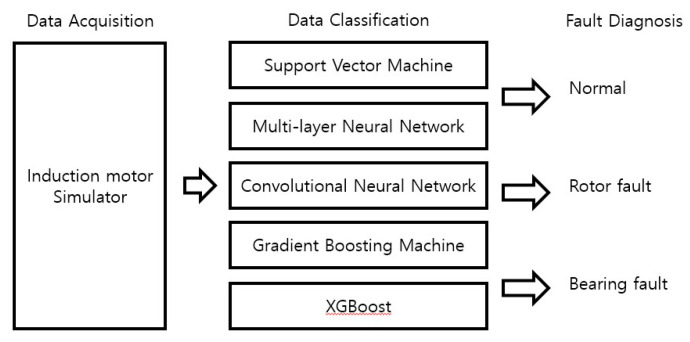
Block diagram of the fault diagnosis algorithm for an induction motor.

The proposed fault diagnosis algorithm is shown in Figure 5; it as constructed using data acquisition and classification steps. In the data acquisition step, the vibration signals from the induction motor are obtained using a vibration sensor and stored as digital data. In the data classification step, the obtained vibration signals are classified into normal, rotor fault, and bearing fault data using the SVM, MNN, CNN, GBM, and XGBoost models.

### 3.1. Support Vector Machine

**Figure 6:**
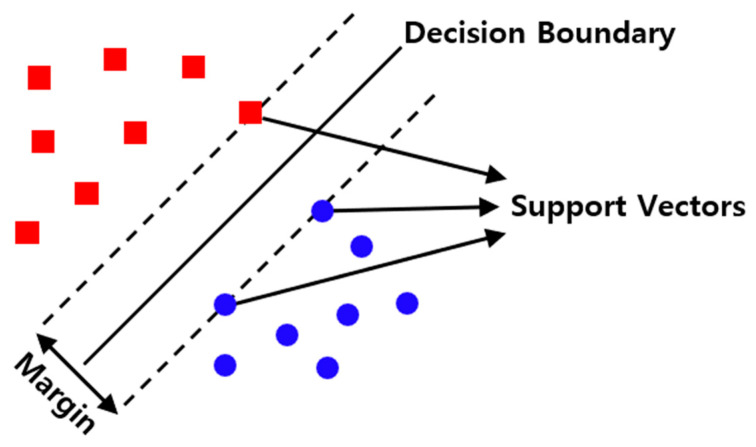
Support vector machine architecture.

SVM is a machine learning model that performs the linear classification of different data classes. It can learn even with a small number of samples and has excellent generalizability [14]. The SVM structure is shown in Figure 6 [15]. The hyperplane is the decision boundary that linearly classifies the data; the support vectors are the data closest to the hyperplane. The margin is the distance between the support vector and hyperplane [16].

The hyperplane in the SVM is expressed as follows:

(1) $$d\left(x\right)=wx_{i}+b,$$

where *x_i_* is the input data, w is the weight vector vertical to the hyperplane, and b is the bias. The length of the margin is calculated using the following equation:

(2) $$\mathrm{margin}=\frac{2}{\left\|w\right\|}+C\sum_{i=1}^{n}\xi_{i}.$$

where $\xi_{i}$ is a slack variable added to adjust the misclassification rate when the data cannot be linearly classified in the SVM, and C is a user-defined parameter [17]. The higher the C value, the lower the permitted degree of misclassification.

In an SVM, a hyperplane that maximizes the margin size should be observed [18]. To this end, we determined the minimum value of *w*. This is an optimization problem, and the constraint and objective function are given by Equations (1) and (2), respectively. The Lagrange multiplier method was used for optimization.

The Lagrange multiplier method obtains the solution of a variable, the partial differentiation value of which is zero for all variables, from the equation that subtracts the value of constraints multiplied by a new variable *α* in the objective function. The final determination function of the SVM obtained using the Lagrange multiplier method is expressed as follows [19,20]:

(3) $$f\left(x\right)=\sum_{i=1}^{N}\alpha_{i}y_{i}K\left(x_{i},\;x\right)+b,$$

where *α_i_* is the Lagrange multiplier, *y_i_* is the output data, *N* is the number of samples in the training data, and *K*() is the kernel.

The kernel is used to map data onto higher dimensions in nonlinear classifications, where data cannot be linearly classified. In this study, we used a radial bias function (RBF) kernel. The RBF kernel is expressed as follows [21,22]:

(4) $$K\left(x_{i},\;x\right)=\exp(\frac{-\|x_{i}-x{\|}^{2}}{2\gamma^{2}}),$$

where γ is a user-defined parameter that controls the flexibility of the decision boundary.

### 3.2. Multilayer Neural Network

**Figure 7:**
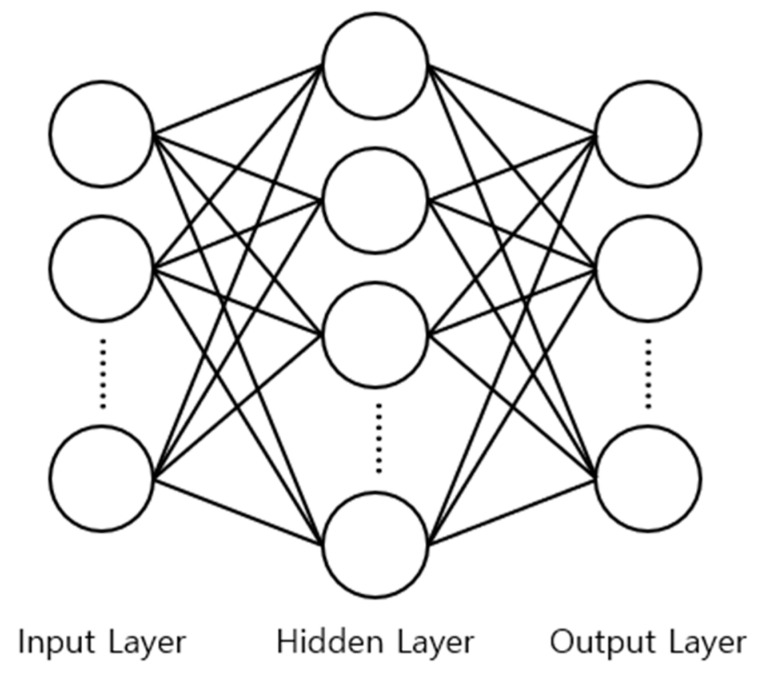
Multilayer neural network architecture.

An MNN is a neural network that complements a single-layer perceptron that cannot be nonlinearly classified because it is exclusively composed of linear functions. MNNs can learn the input and output relationship of nonlinear systems by adjusting weights [23,24]. The MNN structure is shown in Figure 7 [25,26]. One or more hidden layers exist between the input and output layers, and every node in each layer is connected to every node in the adjacent layers [27,28].

An MNN learns via feedforward and backpropagation methods. The feedforward method computes the output using the weight, bias, and activation functions, while the backpropagation method computes the loss function using the neural network output obtained by applying the feedforward method and actual values. Then, the weight is updated in the direction of the loss function gradient reduction using a learning algorithm [29]. In the feedforward method, the input and output values of each node can be expressed as follows [30]:

(5) $$x_{j}=\sum_{i}y_{i}w_{ij}$$

and

(6) $$y_{j}=f(x_{j}+\theta_{j}),$$

where *x_i_* is the input value of node *j*, *w_ij_* is the weight of the output value *y_i_* from node *i* toward node *j* in the previous layer, *y_j_* is the output value of node *j*, *f*() is the activation function, and *θ* is the bias.

In this study, the MNN employed the rectified linear unit (ReLU) activation function in the hidden layer and the SoftMax activation function in the output layer. The ReLU function can alleviate the gradient vanishing problem, unlike the sigmoid and tanh functions [31]. The ReLU function is expressed as follows [32]:

(7) $$f\left(x\right)=\{\begin{array}{c} 0,\;x\leq 0\;\\ x,x>0\; \end{array},$$

where *x* is the input value.

The SoftMax activation function converts the output of the previous layer into the probability of each class in a multiclass classification problem. The SoftMax function is expressed as follows [33,34]:

(8) $$y_{k}=\frac{e^{a_{k}}}{\sum_{i=1}^{n}e^{a_{i}}},$$

where is the number of neurons in the output layer, and *y_k_* is the *k*th output. The numerator is the exponential function of input *a_k_*, and the denominator is the sum of the exponential functions of all input data.

Adam was employed as the optimization algorithm. Adam is an optimization technique that combines the root mean square propagation (RMSProp) technique, which controls the learning rate according to parameter changes, and the momentum technique, which prevents zigzagging of the search path during learning. The formulation is given as follows [35]:

(9) $$m_{0}=0,\;v_{0}=0,\;t=0,$$

(10) $$m_{t}=\beta_{1}m_{t-1}+\left(1-\beta_{1}\right)\nabla_{\theta }f_{t}(\theta_{t-1}),$$

(11) $$v_{t}=\beta_{2}v_{t-1}+\left(1-\beta_{2}\right)\nabla_{\theta }f_{t}(\theta_{t-1}),$$

(12) $$\hat{m_{t}}=\frac{m_{t}}{1-\beta_{1}^{t}},$$

(13) $$\hat{v_{t}}=\frac{v_{t}}{1-\beta_{2}^{t}},$$

and

(14) $$w_{t+1}=w_{t}-\eta \frac{\hat{m_{t}}}{\sqrt{\hat{v_{t}}}+\varepsilon },$$

Where *m*_0_ and *v*_0_ are the first and second moment vectors initialized as zero, respectively; *t* is the time step; $\beta_{1}$ and $\beta_{2}$ are the exponential decay rates for the moment estimates (default values are $\beta_{1}$ = 0.9 and $\beta_{2}$ = 0.999); $\nabla_{\theta }f_{t}\left(\theta_{t-1}\right)$ is the network gradient; η is the learning rate; and ε is a value introduced to prevent the denominator from becoming zero (default value is $10^{-8}$).

### 3.3. Convolutional Neural Network

**Figure 8:**
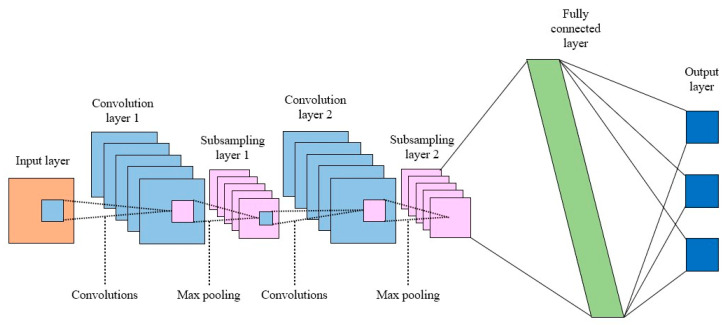
Convolutional neural network architecture.

A CNN extracts features from multidimensional data and then identifies and classifies patterns. The CNN structure is shown in Figure 8 [36]. In Figure 8, the convolution layer performs a convolve operation with the input data using a kernel. Then, it outputs an output feature map using an activation function [37]. The kernel size can be set by the user. The subsampling layer reduces the dimensions of the feature map output from the convolution layer. Note that the size of the feature map can be reduced to reduce computational costs so only the necessary features are extracted [38]. The feature map output by the convolutional and pooling layers is transformed into one dimension and then input to a fully connected layer, and then output as a predicted value through an activation function.

In a CNN, the feature map output by the *L*th convolutional layer is expressed as follows:

(15) $$y_{m}^{l}=f(\sum_{n}x_{n}^{l-1}*k_{n,\;m}^{l}+b_{m}^{l}),$$

where $y_{m}^{l}$ is the *m*th output feature map of the *l*th layer, and $x_{n}^{l-1}$ is the *n*th input feature map. In addition, $k_{n,\;m}^{l}$ is the *m*th kernel corresponding to the *n*th input feature map, and *b* is the bias of the *m*th kernel. *f()* is the activation function, and * is the convolution operation. In this study, the ReLU function was used as an activation function in the convolution layer of the CNN model, and the SoftMax function was used as the activation function of the output layer. The Adam optimizer was also used.

### 3.4. Gradient Boosting Machine

A GBM model is a boosting method that sequentially learns models in an ensemble technique that combines multiple decision trees and is used for classification and regression problems [39]. The learning process of the GBM can be described in five steps. First, the base model is initialized with a constant value. In the second step, the residual is calculated by calculating the partial derivative with the training dataset and the loss function. In the third step, training data and the residual are used to proceed with the basic model’s learning. Using the trained basic model obtained in step three, the optimal coefficients for the loss function are calculated and the model is updated by adding the learning rate (step four). Finally, steps two through four are repeated. Equations (16)–(19) represent the GBM learning process [40].

(16) $$F_{0}\left(x\right)=\arg\underset{\alpha }{\min}\sum_{i=1}^{N}L(y_{i},\;\alpha ),$$

(17) $$r_{im}=-{\left[\frac{\partial L\left(y_{i},\;\;F\left(x_{i}\right)\right)}{\partial F\left(x_{i}\right)}\right]}_{F\left(x\right)=F_{m-1}\left(x\right)},$$

(18) $$\gamma_{m}=arg\;\underset{\gamma }{\min}\sum_{i=1}^{N}L(y_{i},\;F_{m-1}\left(x_{i}\right)+\gamma g_{m}\left(x_{i}\right)),$$

and

(19) $$F_{m}\left(x\right)=F_{m-1}\left(x\right)+v\gamma_{m}g_{m}(x).$$

where *L* is the loss function, $F_{0}\left(x\right)$ is the initial model, $r_{im}$ is the residual, $g_{m}$ is the tree model of the *m*th learning, $\gamma_{m}$ is the optimal coefficient to update the model, v is the learning rate (default value is 0.1), and $F_{m}$ is a GBM model trained m times.

**Figure 9:**
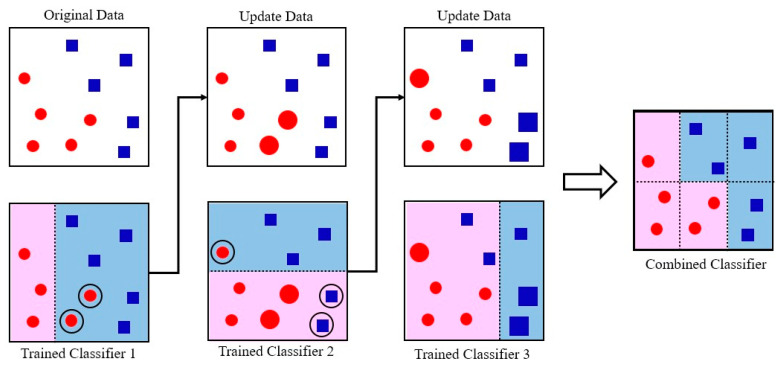
Learning structure of the gradient boosting machine.

Figure 9 shows the GBM learning process used to create a classifier with high accuracy while learning by complementing the weaknesses of the previous model [41]. In Figure 9, red circles and blue squares are different classes of data, while pink and blue zones represent areas where the data are separated.

### 3.5. XGBoost

XGBoost is a machine learning model that complements the GBM model’s vulnerability to overfitting and is used in classification and regression problems. The difference between XGBoost and GBM is that XGBoost controls the complexity of the tree by adding a regularization term to the loss function [42]. In XGBoost, the loss function to which the regularization term is added is expressed as follows [43]:

(20) $$L=\sum_{i=1}^{n}L\left(y,\;F\left(x_{i}\right)\right)+\sum_{m=1}^{m}\Omega \left(g_{m}\right),where\;\Omega \left(g\right)=\gamma T+\frac{1}{2}\lambda \|w{\|}^{2},$$

In Equation (20), Ω is the regularization term added from the loss function of the GBM, which penalizes the complexity of the model; *g_m_* is the *m*th tree; *T* is the number of terminal nodes in the tree; and *w* is the weight of each terminal node. γ and λ are regular term constants. Larger γ values produce simpler trees. If the γ and λ values are set to 0, XGBoost becomes a GBM.

### 3.6. Stratified K-Fold Cross Validation

Stratified K-fold cross validation was used to evaluate the SVM, MNN, CNN, GBM and XGBoost models. Stratified K-fold cross validation is a statistical method used to evaluate learning models that prevents overfitting and adjusts the model parameters when the available data are limited [44,45].

**Figure 10:**
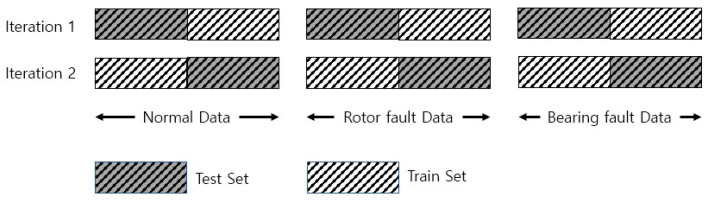
Stratified two-fold cross-validation method.

Stratified K-fold cross validation first divides the data into k groups. One of the segmented data groups is then used for model validation, and the remaining groups are used to train the model. This process is repeated k times, and the average value of the obtained results is used as the verification value. When performing stratified K-fold cross validation, the proportions of the classes constituting the training and verification data must be equal. In addition, all data should only be used once for model validation [46]. In this study, K was set to 2, as in Figure 10.

### 3.7. GUI for Fault Diagnosis in Induction Motor

**Figure 11:**
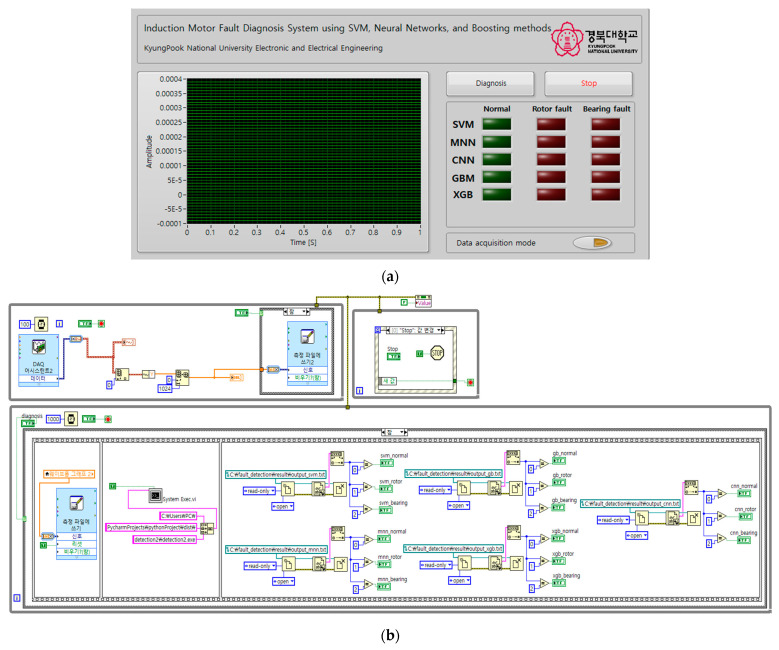
Graphical user interface of fault diagnosis in induction motor. (**a**) Front panel of LabView program for fault diagnosis in induction motor graphical user interface. (**b**) Front panel of LabView program for fault diagnosis in induction motor graphical user interface.

The GUI for the proposed induction motor fault diagnosis technique is shown in Figure 11. The GUI was implemented using LabView (National Instruments, TX, USA). In Figure 11a, the waveform graph on the left shows the vibration data obtained from the induction motor in real time, where the horizontal axis represents time, and the vertical axis represents amplitude. The block diagram of the fault diagnosis GUI implemented through LabView is shown in Figure 11b.

The diagnosis button was used to perform fault diagnosis on the induction motor. When the diagnosis button is pressed, 1024 data samples were extracted from the real-time vibration data and input to the SVM, MNN, CNN, GBM, and XGBoost models to perform fault diagnosis. The stop button terminated the fault diagnosis system. LEDs displayed the fault diagnosis results for the induction motor. 

The button located below the LED as used to acquire vibration data from the induction motor. When this button was pressed, the vibration data were collected and stored in an Excel file. Pressing the button again stopped the data acquisition process.

## 4. Results and Discussion

A The proposed method was experimentally evaluated. The vibration data of the normal, rotor fault, and bearing fault states were recorded, and the acquired data were used to train each model and verify the corresponding diagnostic accuracy. There were 1240 datasets for each induction motor state, and each dataset comprised 1024 data samples.

The SVM, MNN, CNN, and GBM models were implemented using the Python scikit-learn library; the XGBoost model was implemented using the Python XGBoost library. The equation used to obtain the fault diagnosis accuracy is expressed as follows:

(21) $$\mathrm{Accuracy}=\frac{NAD}{NAD+NMD}\times 100,$$

where *NAD* is the number of accurately classified data points, and *NMD* is the number of misclassified data points.

Stratified K-fold cross validation was employed to evaluate each model, where K was set to two. Thus, for each iteration of model training, a total of 1860 datasets were used, and 620 datasets were assigned from each data class. Similarly, verification used a total of 1860 datasets by assigning 620 datasets to each data class.

Table 2 lists the fault diagnosis accuracy and average accuracy for each induction motor state obtained by repeated model learning and verification for the cross-validation of the six SVM models with different γ values. As can be seen, the diagnostic accuracy differed by 6% depending on the data allocated for model training and validation.

Table 3 shows the results of two-fold cross validation for the SVM models obtained using the average values of the training and verification results in Table 2. The SVM model with a γ value of one achieved accuracies of 82.41%, 75.88%, and 100%bfor the normal, rotor fault, and bearing fault states, respectively, with an average accuracy of 86.10%. These results represent the lowest accuracy among all considered models. The SVM model with a γ value of two achieved accuracies of 98.46%, 99.59%, and 100% for the normal, rotor fault, and bearing fault states, respectively, with an average accuracy of 99.35%. These results represent better diagnostic performance than the model with a γ value of one. The SVM model with a γ value of three achieved accuracies of 99.75%, 100%, and 100% for the normal, rotor fault, and bearing fault states, respectively, with an average accuracy of 99.91%. The SVM models with γ values of four and five achieved accuracies of 99.91%, 100%, and 100% for the normal, rotor fault, and bearing fault states, respectively, with an average accuracy of 99.97%. Compared with the SVM model with a γ value of three, these models exhibited a slight improvement; however, they could not obtain 100% diagnostic accuracy for the normal state. Finally, the SVM models with a γ value of six achieved an accuracy of 100% for all states, thereby achieving the best diagnostic accuracy.

The MNN comprised two hidden layers. The numbers of nodes in the first and second hidden layers ere 128 and 64, respectively. In addition, the ReLU function was used as the activation function of the hidden layer, and the number of epochs was set to 250 using the Adam optimization algorithm.

Table 4 shows the fault diagnosis accuracy and average accuracy for each induction motor state obtained using iterated model learning and verification in cross-validation of the MNN model, and Table 5 shows the result of two-fold cross-validation of the MNN model obtained from the results given in Table 4.

The CNN comprised two convolutional layers and two max pooling layers. The kernel size was (3, 3), and the number of kernels was set to 128. In the convolutional layers, the ReLU function was used as the activation function, and the SoftMax function was used in the output layer. In addition, the Adam optimizer was used, and the number of epochs was set to 100. The data input to the CNN input were converted to (32, 32, 1) form.

Table 6 shows the fault diagnosis accuracy and average accuracy for each induction motor state obtained using iterated model learning and verification in the cross-validation of the CNN model.

Table 7 shows the results of the two-fold cross-validation of the CNN model obtained from the results shown in Table 6.

Table 8 lists the fault diagnosis accuracy and average accuracy for each induction motor state obtained by repeated model learning and verification for the cross-validation of four GBM models with different numbers of trees.

Table 9 lists the results of the two-fold cross-validation for the GBM models obtained by the average values of the training and verification results given in Table 8. The tree max depth of GBM was set to 3, and the learning rate was set to 0.1. As can be seen, the GBM model with 700 trees obtained an accuracy of 98.70% for the normal state, 99.27% for the rotor fault state, and 100% for the bearing fault state, for an average accuracy of 99.32%. The GBM model with 800 trees obtained an accuracy of 98.62% for the normal state, 99.51% for the rotor fault state, and 100% for the bearing fault state, for an average accuracy of 99.37%. The 900-tree GBM model obtained an accuracy of 98.70% for the normal state, 99.51% for the rotor fault state, and 100% for the bearing fault state, for an average accuracy of 99.40%. Thus, the GBM model with 900 trees achieved the best diagnostic performance among the compared GBM models. The GBM model with 1000 trees obtained an accuracy of 98.62% for the normal state, 99.51% for the rotor fault state, and 100% for the bearing fault state, for an average accuracy of 99.37%.

Table 10 lists the fault diagnosis accuracy and average accuracy for each induction motor state obtained by repeated model learning and verification for cross-validation of three XGBoost models with different numbers of trees.

Table 11 lists the two-fold cross-validation results for the XGBoost models obtained according to the average values of the training and verification results given in Table 10. The tree max depth of XGBoost was set to 4 and the learning rate to 0.2. As shown, the XGBoost model with 10 trees achieved an accuracy of 71.69% for the normal state, 64.19% for the rotor fault state, and 100% for the bearing fault state, for an average accuracy of 78.62%. The XGBoost model with 50 trees obtained an accuracy of 80.32% for the normal state, 73.31% for the rotor fault state, and 100% for the bearing fault state, for an average accuracy of 84.54%. The 100-tree XGBoost model obtained an accuracy of 98.22% for the normal state, 99.27% for the rotor fault state, and 100% for the bearing fault state, for an average accuracy of 99.16%. The XGBoost model with 700 trees obtained an accuracy of 99.11% for the normal state, 99.76% for the rotor fault state, and 100% for the bearing fault state, for an average accuracy of 99.62%; these models achieved the best diagnostic performance among the considered XGBoost models. The XGBoost model with 1000 trees obtained an accuracy of 99.03% for the normal state, 98.70% for the rotor fault state, and 100% for the bearing fault state, for an average accuracy of 99.25%.

Table 12 compares the diagnostic accuracies obtained through the cross-validation of the optimal SVM, MNN, CNN, GBM, and XGBoost models. As can be seen, the MNN model obtained accuracies of 99.67%, 100%, and 97.49% for the normal, rotor fault, and bearing fault states, respectively, with an average accuracy of 99.05%. The GBM model realized accuracies of 98.70%, 99.51%, and 100% for the normal, rotor fault, and bearing fault states, respectively, with an average accuracy of 99.40%. The XGBoost model obtained accuracies of 99.11%, 99.76%, and 100% for the normal, rotor fault, and bearing fault states, respectively, with an average accuracy of 99.62%. The optimal SVM and CNN models demonstrated 100% accuracy for all states; thus, the average accuracy was 100%. Therefore, the optimal SVM and CNN models outperformed the MNN, GBM, and XGBoost models.

**Figure 12:**
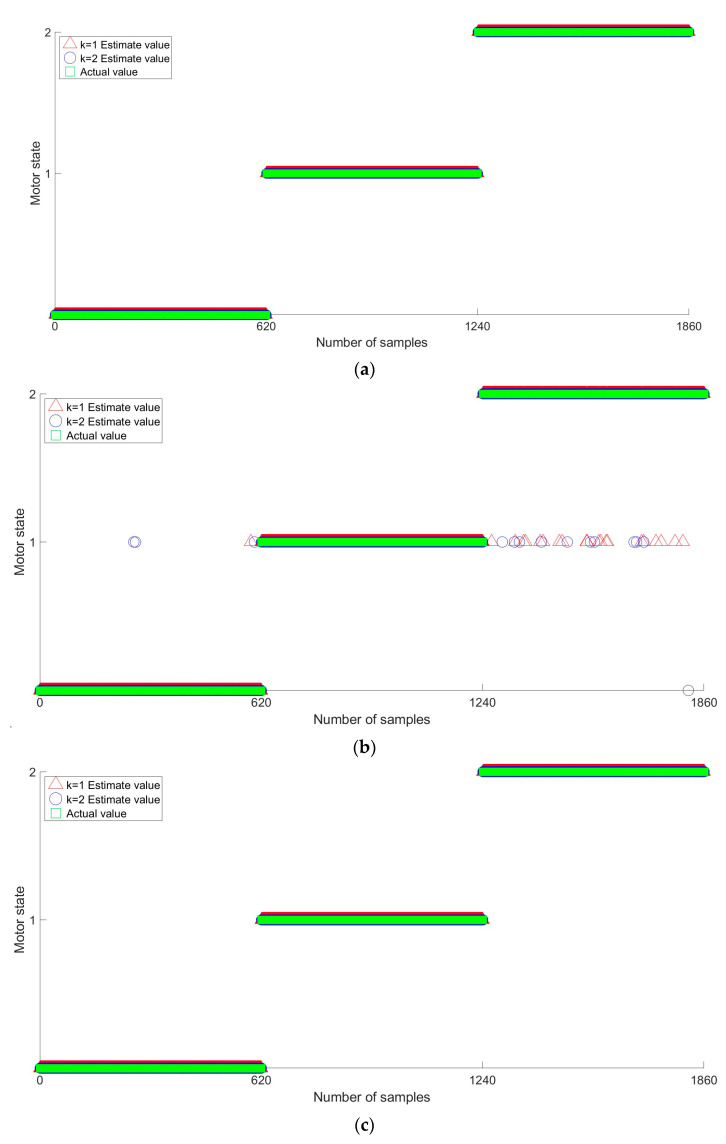

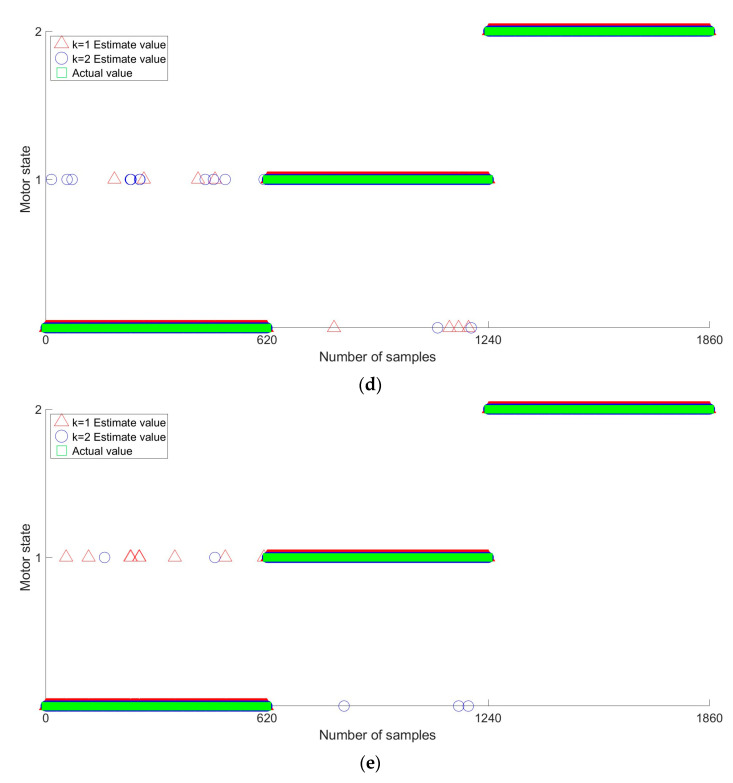
Results of the fault diagnosis using the optimal SVM, MNN, CNN, GBM, and XGBoost models. (**a**) Fault diagnosis result of the optimal support vector machine (SVM) model. (**b**) Fault diagnosis result of multilayer neural network (MNN) model. (**c**) Fault diagnosis result of convolutional neural network (CNN) model. (**d**) Fault diagnosis result of the optimal gradient boosting machine (GBM) model. (**e**) Fault diagnosis result of the optimal XGBoost model.

The graphs in Figure 12 show the failure diagnosis results obtained via the cross-validation of the SVM, MNN, CNN, GBM, and XGBoost models. In the graph, the horizontal and vertical axes represent the number of data samples and the induction motor state, respectively. On the vertical axis, zero, one, and two represent the normal, rotor fault, and bearing fault states, respectively. In addition, k = 1 represents the first iteration in the cross-validation process.

As shown, the SVM model with a γ value of six was 100% for all the states. The MNN model obtained accuracies of 99.67%, 100%, and 97.49% for the normal, rotor fault, and bearing fault states, respectively. The CNN model obtained accuracies of 100% for all states. The GBM model with 900 trees obtained accuracies of 98.70%, 99.51%, and 100% for the normal, rotor fault, and bearing fault states, respectively. The XGBoost model with 700 trees obtained accuracies of 99.11%, 99.76%, and 100% for the normal, rotor fault, and bearing fault states, respectively. Among the five compared models, we found that the SVM and CNN models outperformed the MNN, GBM and XGBoost models.

In addition, we confirmed that the SVM, GBM, and XGBoost models could misdiagnose the normal state as a rotor fault (and vice versa). Similarly, the MNN model could misdiagnose the normal state as a rotor fault and a bearing fault as either a rotor fault or the normal state.

Table 13 shows the computation time for the test data in each iteration during the cross-validation of the SVM, MNN, CNN, GBM, and XGBoost models. The average value confirmed that the XGBoost model was the fastest at 0.05009 s, and the SVM model was the slowest at 1.65048 s. We confirmed the diagnosis accuracy in Table 12 and the computation speed in Table 13; we found that SVM and CNN had the best diagnostic accuracy and XGBoost had the fastest computation speed.

**Figure 13:**
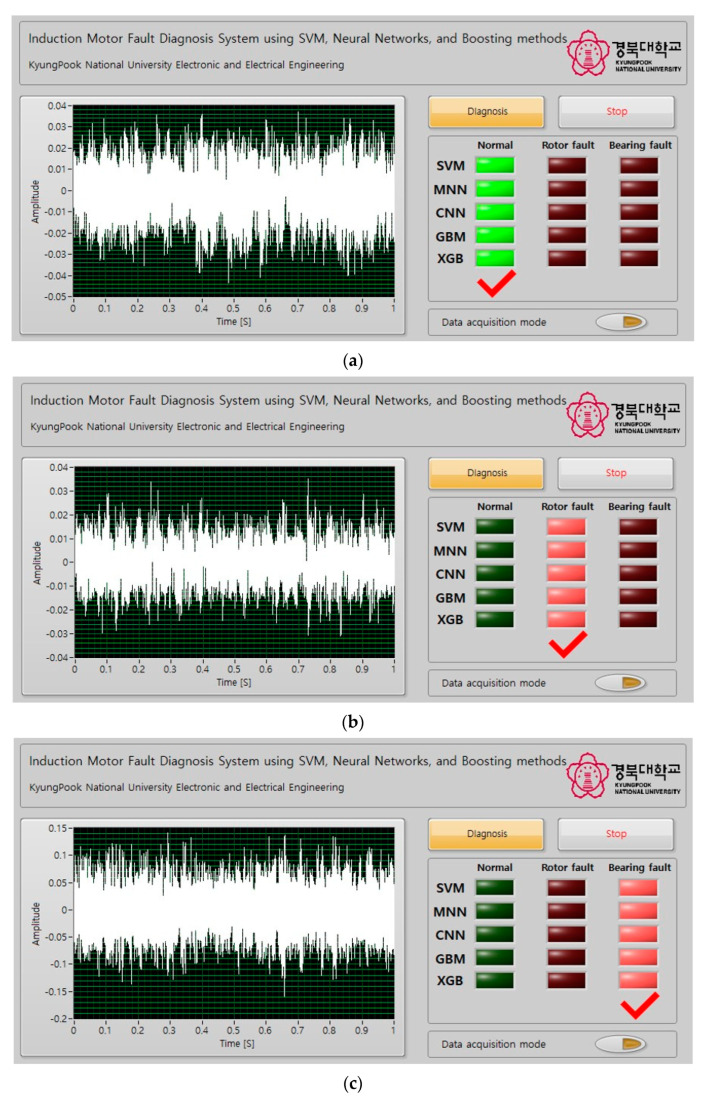
Fault diagnosis and results of induction motor using graphical user interface. (**a**) Results of normal state induction motor fault diagnosis using graphical user inter-face. (**b**) Results of rotor fault state induction motor fault diagnosis using graphical user interface. (**c**) Results of bearing fault state induction motor fault diagnosis using graphical user interface.

Figure 13a–c show the induction motor fault diagnosis results using the GUI. As can be seen, the LED of the GUI was turned on in accordance with the state of the induction motor. These results verified that the implemented GUI can be used to accurately diagnose the state of an induction motor.

## 5. Conclusions

In this study, induction motor fault diagnosis was performed using SVM, MNN, CNN, GBM, and XGBoost models. In the experiment, an induction motor simulator running in normal, rotor fault, and bearing fault states was fabricated, and real-time vibration data were acquired to perform fault diagnosis. Then, a GUI for the proposed fault diagnosis method was implemented.

The SVM consisted of six models with C = 1 and γ set to 1, 2, 3, 4, 5, and 6. The values for parameters C and γ were determined by the user. The MNN model used the ReLU activation function for the two hidden layers; the Adam optimizer was used as the optimization algorithm with 250 epochs. The CNN model comprised two convolutional layers and pooling layers. In the convolutional layers, 128 kernels of size (3, 3) were used, and the ReLU function was used as the activation function. In addition, max pooling was utilized in the pooling layers. In the output layer, the SoftMax function was used as the activation function, and the Adam optimizer was employed. The data input to the CNN were converted to (32, 32, 1) form, and the number of epochs was set to 100. Four GBM models with 700, 800, 900, and 1000 trees were compared; we set the tree max depth to 3 and the learning rate to 0.1. Five XGBoost models with 10, 50, 100, 700, and 1000 trees were compared; we set the tree max depth to 4 and the learning rate to 0.2. Subsequently, the accuracy and calculated speed of the models used for fault diagnosis were evaluated using stratified two-fold cross-validation.

Among the SVM models, the model with a γ value of one achieved accuracies of 82.41%, 75.88%, and 100% for the normal, rotor fault, and bearing fault states, respectively, with an average accuracy of 86.10%. The SVM model with a γ value of two achieved accuracies of 98.46%, 99.59%, and 100% for the normal, rotor fault, and bearing fault states, respectively, with an average accuracy of 99.35%. In addition, the SVM model with a γ value of three achieved accuracies of 99.75%, 100%, and 100% for the normal, rotor fault, and bearing fault states, respectively, with an average accuracy of 99.91%. The models with γ values of four and five exhibited accuracies of 99.91%, 100%, and 100% for the normal, rotor fault, and bearing fault states, respectively, with an average accuracy of 99.97%. The model with a γ value of six achieved 100% diagnostic accuracy for all states. By comparing the fault diagnosis performance of the six SVM models, we confirmed that the error in the diagnosis exhibited a difference of approximately 14% due to the set parameter values. With the MNN model, the accuracy values obtained for the normal, rotor fault, and bearing fault states were 99.67%, 100%, and 97.49%, respectively, with an average accuracy of 99.05%. The CNN model obtained 100% diagnostic accuracy for all states.

Among the compared GBM models, we found that the model with 700 trees achieved accuracies of 98.70%, 99.27%, and 100% for the normal, rotor fault, and bearing fault states, respectively, with an average accuracy of 99.32%. The GBM model with 800 trees achieved accuracies of 98.62%, 99.51%, and 100% for the normal, rotor fault, and bearing fault states, respectively, showing an average accuracy of 99.37%. The model with 900 trees obtained accuracies of 98.70%, 99.51%, and 100% for the normal, rotor fault, and bearing fault states, respectively, with an average accuracy of 99.40%. The model with 1000 trees exhibited accuracies of 98.62%, 99.51%, and 100% for the normal, rotor fault, and bearing fault states, respectively, with an average accuracy of 99.40%. By comparing the fault diagnosis performance of the four GBM models, we confirmed that the model with 900 trees outperformed the other GBM models. Among the compared XGBoost models, we found that the model with 10 trees achieved accuracies of 71.69%, 64.19%, and 100% for the normal, rotor fault, and bearing fault states, respectively, with an average accuracy of 78.62%. The XGBoost model with 50 trees achieved accuracies of 80.32%, 73.31%, and 100% for the normal, rotor fault, and bearing fault states, respectively, showing an average accuracy of 84.54%. The model with 100 trees obtained accuracies of 98.22%, 99.27%, and 100% for the normal, rotor fault, and bearing fault states, respectively, with an average accuracy of 99.16%. The model with 700 trees exhibited accuracies of 99.11%, 99.76%, and 100% for the normal, rotor fault, and bearing fault states, respectively, with an average accuracy of 99.62%. The model with 1000 trees exhibited accuracies of 99.03%, 98.70%, and 100% for the normal, rotor fault, and bearing fault states, respectively, with an average accuracy of 99.25%. By comparing the fault diagnosis performance of the five XGBoost models, we confirmed that the model with 700 trees outperformed the other XGBoost models.

During the cross-validation of the compared SVM, MNN, CNN, GBM, and XGBoost models, the computation time for the test data was measured in each iteration. Here, we found that the XGBoost model was the fastest (0.05009 s), followed by the GBM model (0.09000 s), MNN model (0.09079 s), CNN model (0.48610 s), and SVM model (1.65048 s). We confirmed that SVM and CNN had the best diagnostic accuracy, and XGBoost had the fastest computation speed.

In addition, by performing fault diagnosis of the induction motor using the implemented GUI, we verified that induction motors in the normal, rotor fault, and bearing fault states were accurately diagnosed. In the future, we will use machine learning models other than those used in this study to perform fault diagnosis and apply them to a real environment for further comparison and analysis.

## Figures and Tables

**Table 1:** Specifications of the vibration sensor used for data acquisition and fault diagnosis.

Model Number	603C01
Measurement range	490 m/s^2^
Frequency range	0.5–10,000 Hz
Transverse sensitivity	≤7%
Temperature range	−54 °C–121 °C

**Table 2:** Diagnostic accuracy of support vector machine for cross-validation.

Iteration	C, γ	Normal	Rotor Fault	Bearing Fault	Average
1	1, 1	85.64%	77.09%	100%	87.58%
	1, 2	99.19%	99.67%	100%	99.68%
	1, 3	100%	100%	100%	100%
	1, 4	100%	100%	100%	100%
	1, 5	100%	100%	100%	100%
	1, 6	100%	100%	100%	100%
2	1, 1	79.19%	74.67%	100%	84.62%
	1, 2	97.74%	99.51%	100%	99.08%
	1, 3	99.51%	100%	100%	99.83%
	1, 4	99.83%	100%	100%	99.94%
	1, 5	99.83%	100%	100%	99.94%
	1, 6	100%	100%	100%	100%

**Table 3:** Diagnostic accuracy of support vector machine through two-fold cross validation.

C, γ	Normal	Rotor Fault	Bearing Fault	Average
1, 1	82.41%	75.88%	100%	86.10%
1, 2	98.46%	99.59%	100%	99.35%
1, 3	99.75%	100%	100%	99.91%
1, 4	99.91%	100%	100%	99.97%
1, 5	99.91%	100%	100%	99.97%
1, 6	100%	100%	100%	100%

**Table 4:** Diagnostic accuracy of multilayer neural network for cross-validation.

Iteration	Normal	Rotor Fault	Bearing Fault	Average
1	99.83%	100%	99.67%	98.87%
2	99.51%	100%	98.22%	99.24%

**Table 5:** Diagnostic accuracy of multilayer neural network through two-fold cross-validation.

Normal	Rotor Fault	Bearing Fault	Average
99.67%	100%	97.49%	99.05%

**Table 6:** Diagnostic accuracy of convolutional neural network for cross-validation.

Iteration	Normal	Rotor Fault	Bearing Fault	Average
1	100%	100%	100%	100%
2	100%	100%	100%	100%

**Table 7:** Diagnostic accuracy of convolutional neural network through two-fold cross validation.

Normal	Rotor Fault	Bearing Fault	Average
100%	100%	100%	100%

**Table 8:** Diagnostic accuracy of gradient boosting machine for cross-validation.

Iteration	Number of Trees	Normal	Rotor Fault	Bearing Fault	Average
1	700	99.03%	99.19%	100%	99.40%
	800	99.03%	99.51%	100%	99.51%
	900	99.35%	99.35%	100%	99.56%
	1000	99.19%	99.35%	100%	99.51%
2	700	98.38%	99.35%	100%	99.24%
	800	98.22%	99.51%	100%	99.24%
	900	98.06%	99.67%	100%	99.24%
	1000	98.06%	99.67%	100%	99.24%

**Table 9:** Diagnostic accuracy of gradient boosting machine through two-fold cross-validation.

Number of Trees	Normal	Rotor Fault	Bearing Fault	Average
700	98.70%	99.27%	100%	99.32%
800	98.62%	99.51%	100%	99.37%
900	98.70%	99.51%	100%	99.40%
1000	98.62%	99.51%	100%	99.37%

**Table 10:** Diagnostic accuracy of XGBoost for cross-validation.

Iteration	Number of Trees	Normal	Rotor Fault	Bearing Fault	Average
1	10	72.58%	61.93%	100%	78.17%
	50	83.87%	74.68%	100%	86.18%
	100	97.25%	99.19%	100%	98.81%
	700	98.54%	100%	100%	99.51%
	1000	99.19%	98.70%	100%	99.30%
2	10	70.80%	66.45%	100%	79.08%
	50	76.77%	71.94%	100%	82.90%
	100	99.19%	99.35%	100%	99.51%
	700	99.67%	99.51%	100%	99.73%
	1000	98.87%	98.70%	100%	99.19%

**Table 11:** Diagnostic accuracy of XGBoost through two-fold cross-validation.

Number of Trees	Normal	Rotor Fault	Bearing Fault	Average
10	71.69%	64.19%	100%	78.62%
50	80.32%	73.31%	100%	84.54%
100	98.22%	99.27%	100%	99.16%
700	99.11%	99.76%	100%	99.62%
1000	99.03%	98.70%	100%	99.25%

**Table 12:** Diagnostic accuracy of optimal SVM, MNN, CNN, GBM, and XGBoost models through stratified two-fold cross-validation.

Method of Classification	Normal	Rotor Fault	Bearing Fault	Average
SVM	100%	100%	100%	100%
MNN	99.67%	100%	97.49%	99.05%
CNN	100%	100%	100%	100%
GBM	98.70%	99.51%	100%	99.40%
XGBoost	99.11%	99.76%	100%	99.62%

**Table 13:** Computation time for test data during cross-validation of SVM, MNN, CNN, GBM, and XGBoost models.

	Iteration 1	Iteration 2	Average
MNN	0.11637 s	0.06522 s	0.09079 s
SVM	1.61548 s	1.68549 s	1.65048 s
GBM	0.09001 s	0.08999 s	0.09000 s
XGBoost	0.05018 s	0.05001 s	0.05009 s
CNN	0.51737 s	0.45484 s	0.48610 s

## Data Availability

Not applicable.

## Abbreviations

The following abbreviations are used in this manuscript:

SVM	Support vector machine
MNN	Multilayer neural network
CNN	Convolutional neural network
GBM	Gradient boosting machine
GUI	Graphical user interface
RBF	Radial bias function
ReLU	Rectified linear unit
RMSProp	Root mean square propagation
NAD	Number of accurately classified data points
NMD	Number of misclassified data points
